# Bending of Protonema Cells in a Plastid Glycolate/Glycerate Transporter Knockout Line of *Physcomitrella patens*

**DOI:** 10.1371/journal.pone.0118804

**Published:** 2015-03-20

**Authors:** Jin Nakahara, Katsuaki Takechi, Fumiyoshi Myouga, Yasuko Moriyama, Hiroshi Sato, Susumu Takio, Hiroyoshi Takano

**Affiliations:** 1 Graduate School of Science and Technology, Kumamoto University, Kurokami, Kumamoto 860–8555, Japan; 2 Gene Discovery Research Group, RIKEN Center for Sustainable Resource Science (CSRS), Yokohama, Kanagawa 230–0045, Japan; 3 Faculty of Science, Kumamoto University, Kurokami, Kumamoto 860–8555, Japan; 4 Center for Marine Environment Studies, Kumamoto University, Kurokami, Kumamoto 860–8555, Japan; 5 Institute of Pulsed Power Science, Kumamoto University, Kumamoto 860–8555, Japan; Institute of Genetics and Developmental Biology, Chinese Academy of Sciences, CHINA

## Abstract

*Arabidopsis* LrgB (synonym PLGG1) is a plastid glycolate/glycerate transporter associated with recycling of 2-phosphoglycolate generated via the oxygenase activity of ribulose-1,5-bisphosphate carboxylase/oxygenase (RuBisCO). We isolated two homologous genes (*PpLrgB1* and *B2*) from the moss *Physcomitrella patens*. Phylogenetic tree analysis showed that PpLrgB1 was monophyletic with LrgB proteins of land plants, whereas PpLrgB2 was divergent from the green plant lineage. Experiments with PpLrgB–GFP fusion proteins suggested that both PpLrgB1 and B2 proteins were located in chloroplasts. We generated *PpLrgB* single (∆B1 and ∆B2) and double (∆B1/∆B2)-knockout lines using gene targeting of *P*. *patens*. The ∆B1 plants showed decreases in growth and photosynthetic activity, and their protonema cells were bent and accumulated glycolate. However, because ∆B2 and ∆B1/∆B2 plants showed no obvious phenotypic change relative to the wild-type or ∆B1 plants, respectively, the function of PpLrgB2 remains unclear. *Arabidopsis* LrgB could complement the ∆B1 phenotype, suggesting that the function of PpLrgB1 is the same as that of AtLrgB. When ∆B1 was grown under high-CO_2_ conditions, all novel phenotypes were suppressed. Moreover, protonema cells of wild-type plants exhibited a bending phenotype when cultured on media containing glycolate or glycerate, suggesting that accumulation of photorespiratory metabolites caused *P*. *patens* cells to bend.

## Introduction

Photorespiration is essential for the viability of all oxygen-producing photosynthetic organisms (reviewed in [1]). The process commences with generation of 2-phosphoglycolate (2-PG) via the oxygenase activity of ribulose 1,5-biphosphate (RuBP) carboxylase/oxygenase (RuBisCO). After conversion of 2-PG to glycolate by 2-phosphoglycolate phosphatase (PGLP) in the stroma, glycolate is transported to the peroxisome via the cytosol. Glycolate is oxidized to glyoxylate by glycolate oxidase (GOX), and the product is next transaminated to form glycine by serine:glyoxylate (SGT) and glutamate:glyoxylate aminotransferase (GGT) in the peroxisome. Two molecules of glycine are transported to mitochondria and converted therein to one molecule of serine, with release of carbon dioxide (CO_2_) and ammonia. Serine returns to the peroxisome and is changed to glycerate via conversion of glyoxylate to glycine by SGT and hydroxypyruvate reductase (HPR) in that organelle. Finally, glycerate is transported to chloroplasts via the cytosol and phosphorylated by glycerate kinase (GLYK) to form 3-phosphoglycerate (3-PGA), which can enter the Calvin cycle. As photorespiration in plants involves three organelles, plastids, mitochondria, and peroxisomes, in addition to the cytosol, at least 20 transporters are expected to be involved in the core carbon metabolism and associated processes [2]. Of these transporters, one gene family encoding plastid dicarboxylate translocators involved in nitrogen recycling had been identified in addition to discovery of the plastid glycolate/glycerate transporter PLGG1 (synonym AtLrgB) [3,4].

We earlier found that *Arabidopsis* (*At*) *LrgB* (*At1g32080*) corresponded to the gene mutated in three *albino or pale-green* (*apg*) mutants in *Ac/Ds*-tagged lines generated by RIKEN [5]. In the same year, Yang et al. characterized AtLrgB [6]. During continuous observation of seedlings of *atlrgB* mutants growing under short-day conditions, we found that the cotyledons and true leaves of mutant plants exhibited immediate greening, similar to wild-type (WT) plants, after which some parts of the tissues developed a chlorotic cell death phenotype [5]. An amino acid homology search suggested that the C-terminal region of AtLrgB was homologous to that of the bacterial membrane protein LrgB, which is speculated to counter cell death and lysis in bacteria [5, 6]. Although the detailed function of bacterial LrgB remains unclear, both bacterial and chloroplast LrgB are thought to inhibit cell death. Therefore, we named the protein AtLrgB, although the molecular functions thereof were unknown at that time.

As AtLrgB contained 12 putative transmembrane domains, the protein was predicted to be a transporter located in the plastid envelope [7]. Mass spectrometry of chloroplast envelopes confirmed that AtLrgB was located in the inner envelope [8]. Recently, Pick et al. revealed that At1g32080 (AtLrgB) encodes a photorespiratory glycolate/glycerate translocator (PLGG1) of the plastid envelope [4]. Glycolate and glycerate are transported by the same transporter [4, 9]. As expected, the *atlrgB* mutant accumulated glycolate and glycerate in addition to other photorespiratory metabolites, and in vivo and in vitro transport assays confirmed that AtLrgB had a transport function [4].

*Arabidopsis* photorespiratory mutants exhibit phenotypes ranging from severe lethality to minor physiological changes, and the phenotypes are strongly affected by the CO_2_ level (reviewed in [10]). Several mutants with mutations in the *PGLP1* and *GLYK* genes are viable when grown in elevated CO_2_, but exhibit lethality when transferred from high to low CO_2_ conditions. This is the “photorespiratory phenotype” described by Somerville [11]. In contrast, several mutants with mutations in the *GGT1* and *HPR1* genes exhibit retarded growth, but remain viable, when grown in normal air [10]. Also, some mutants in photorespiratory genes do not exhibit photorespiration phenotypes, suggesting that such genes are redundant or function only indirectly in photorespiration [10]. The *atlrgB* mutants showed chlorotic cell death phenotypes, with accumulation of photorespiratory metabolites, when grown in ambient air. However, the phenotype is relatively mild because mutant plants with variegated leaves are nonetheless viable in air. Glycolate is thought to be able to leak through lipid bilayers by slow passive diffusion, as shown by other small organic acids, which may explain the mild phenotype exhibited by *atlrgB* mutants. As with other photorespiratory mutants, the cell death phenotypes of *atlrgB* mutants were suppressed under high-CO_2_ conditions [4].

Many photorespiratory mutants have been isolated from different plant species including *Arabidopsis*, tobacco, rice, and maize, as well as green algae and cyanobacteria (reviewed in [10]), but not bryophytes those diverged from vascular plants early after land colonization [12]. The data show that photorespiration is essential not only for C_3_ and C_4_ plants, but also for green algae and cyanobacteria, growing in ambient air [10]. The moss *Physcomitrella patens* is used as a model plant due to a high frequency of homologous recombination [13] and availability of the entire genome sequence [12]. Similar to other bryophytes, the life cycle of *P*. *patens* is dominated by a haploid gametophyte phase. A spore germinates into chloronema, one type of protonema. Caulonema, the other type of protonema, arises from chloronema cells, and can form gametophores. Both female and male organs form at the apex of the gametophore and, after fertilization, sporophytes develop.

Land plants, including mosses, must be able to deal with variable light intensities because excess light energy channeled into photosynthesis generates reactive oxygen species (ROS), causing photodamage and photoinhibition. The fastest response to high light stress is provided by non-photochemical quenching (NPQ) that is a mechanism dissipating excess energy as heat. The second protection mechanism includes photorespiration, water-water cycle, cyclic electron transport within Photosystem I (PSI) and so on. In vascular plants, NPQ relies on the activity of S subunit of Photosystem II (PSBS), while algae use light-harvesting complex (Lhc)-like polypeptide, LHCSR for NPQ. *P*. *patens* occupies an evolutionary intermediate position between algae and vascular plants, and exhibits high-level NPQ (in contrast to *Arabidopsis*) via both algal-type LHCSR-dependent and plant-type PSBS-dependent mechanisms [14]. This high-level NPQ may influence other photoprotection mechanisms including photorespiration. In the present paper, we explored the *LrgB* gene of the moss and analyzed the phenotypes of mutants generated via gene-targeting techniques.

## Results

### *LrgB* homologous genes in *P. patens*

To isolate *P*. *patens LrgB* genes, the genomic sequence of *P*. *patens* [12] was searched using amino-acid sequences of *AtLrgB* from *A*. *thaliana*. We found two homologous genes in the *P*. *patens* genome, and termed them *PpLrgB1* and *B2*. Phylogenetic analysis showed that the PpLrgB1 protein was monophyletic with LrgB proteins of land plants, whereas PpLrgB2 belonged to a lineage divergent from the green plant lineage (S1 and S2 Figs.). Northern analysis indicated that both *PpLrgB* genes were expressed in the protonemata of *P*. *patens* (S3a Fig.)

The accession numbers used in this study are NP_564388 (*A. thaliana*), XP_003547813.1 (*Glycine max* LrgB1), XP_003516843.1 (G. max LrgB2), NP_001065502 (*Oryza sativa* LrgB1), EEE54674 (*O. sativa* LrgB2), XP_002304362 (*Populus trichocarpa*), CBI31242.3 (*Vitis vinifera* LrgB1), XP_002277191 (*V. vinifera* LrgB2), NP_001151575 (*Zea mays* LrgB1) and NP_001169302.1 (*Z. mays* LrgB2) from land plants, XP_001694486 (*Chlamydomonas reinharditii*), XP_001416011 (*Ostreococcus lucimarinus*), CAL49912 (*Ostreococcus tauri*) and XP_005536986 (*Cyanidioschyzon merolae*) from green and red algae, XP_002180264 (*Phaeodactylum tricornutum* LrgB1), XP_002180004 (*P. tricornutum* LrgB2), XP_002293321 (*Thalassiosira pseudonana* LrgB1) and XP_002296208 (*T. pseudonana* LrgB2) from diatom, NP_126052.1 (*Pyrococcus abyssi*), YP_004423322.1 (*Pyrococcus* sp. NA2), NP_143637.1 (*P. horikoshii*), YP_004763139.1 (*Thermococcus* sp. 4557), YP_002582288 (*Thermococcus* sp. AM4), YP_006425185.1 (*Thermococcus* sp. CL1), ZP_09729779 (*T. litoralis*), YP_002960186.1 (*T. gammatolerans*), YP_002993502.1 (T. *sibiricus*), YP_004072173.1 (*T. barophilus*) and YP_002307911.1 (*T. onnurineus*) from archaea, YP_002422676 (*Methylobacterium chloromethanium*), YP_916520 (*Paracoccus denitrificans*) and YP_001989581 (*Rhodopseudomonas palustris*) from α-proteobacteria, YP_001586009 (*Burkholderia multivorans*), YP_286306 (*Dechloromonas aromatica*), YP_727542 (*Ralstonia eutropha*), YP_297000 (*R. eutropha*), YP_001900461 (*R. pickettii*), ZP_00944293 (*R. solanacearum*) and YP_002297100 (*Rhodospirillum centenum*) from ß-proteobacteria, YP_002850122 (*Citrobacter* sp.), ZP_04534126 (*Escherichia albertii*), ACA77168 (*E. coli*), ZP_04534126 (*Escherichia* sp. 3_2_53FAA), NP_439449 (*Haemophilus influenzae*), ZP_01160777 (*Photobacterium* sp.), YP_610074 (*Pseudomonas entomophila*), YP_001670831 (*P. putida*), YP_002048263 (*Salmonella enterica*), YP_002356917 (*Shewanella baltica*), YP_204867 (*Vibrio fischeri*) and NP_992767 (*Yersinia pestis*) from γ-proteobacteria, and CAB15859 (*Bacillus subtilis*), YP_002861195 (*Clostridium botulinum*), YP_002505749 (*C*. *cellulolyticum*), NP_816796 (*Enterococcus faecalis*), WP_003642478 (*Lactobacillus plantarum* subsp. *Plantarum*), ZP_03168284 (*Ruminococcus lactaris*), A5IPD29 (*Staphylococcus aureus*) and YP_252525 (*S*. *haemolyticus*) from gram-positive bacteria.

### Subcellular localization of *PpLrgBs*

**Fig 1:**
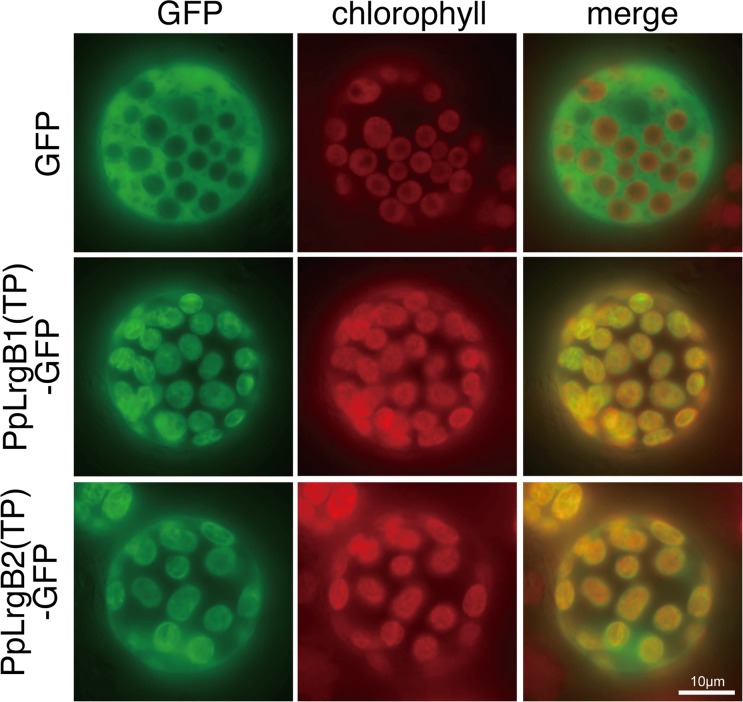
Subcellular locations of GFP fusion proteins. Transient expression of two constructs [PpLrgB1(TP)-GFP and PpLrgB2(TP)-GFP] in *Physcomitrella patens* protoplasts. The empty green fluorescent protein (GFP) vector served as a control. Fluorescent images of GFP and chlorophyll autofluorescence, and merged images, are shown.

The TargetP program predicted that both *PpLrgB1* and *B2* encoded plastid-targeting sequences of 39 and 38 amino acids, respectively, with corresponding scores of 0.87 and 0.51. To explore the subcellular locations of PpLrgB1 and B2, we constructed two plasmids in which the Cauliflower mosaic virus (CaMV) 35S promoter directed expression of the putative transit peptide (TP) fused to green fluorescent protein (GFP). After polyethylene glycol (PEG)-mediated transformation of the plasmids, the GFP fusion proteins, which bore the N-terminal regions of either PpLrgB1 or B2, were observed in chloroplasts of *P*. *patens*, corroborating the computer predictions (Fig. 1).

### Generation of transformants of *P. patens*

We constructed plasmids to generate *PpLrgB*-knockout transformants (S4 Fig.). The 5′ and 3′ genomic regions of the *PpLrgB1* and *B2* genes were amplified via genomic polymerase chain reaction (PCR) and cloned. The neomycin phosphotransferase (*NPTII*) gene, or the zeocin-resistance gene, driven by the CaMV35S promoter and terminated using the CaMV35S polyadenylation signal, was inserted between the 5′ and 3′ genomic regions of *PpLrgB1* or *PpLrgB2*, respectively, followed by PEG-mediated transformation into *P*. *patens*. Southern hybridization experiments were performed to determine plasmid copy number. After *PpLrgB1* gene targeting, line #2 showed a single insertion of the *NPTII* gene (S4c Fig.). Single insertions of the *PpLrgB2* gene were found in two transformants (S4d Fig.). To generate *PpLrgB1/B2* (ΔB1/ΔB2) double-knockout lines, we disrupted the *PpLrgB2* gene in *PpLrgB1* (ΔB1) knockout line #2. Southern hybridization revealed disruption of both *PpLrgB1* and *PpLrgB2* in two lines (S4e Fig.). Reverse transcription (RT)-PCR showed that *PpLrgB* transcripts were not detected in knockout transformants (S3b Fig.).

To compare the functions of the *LrgB* genes of *P*. *patens* and *A*. *thaliana*, stable transformants expressing AtLrgB were generated using the ΔB1-knockout line. First, *AtLrgB* cDNA was cloned between the rice actin promoter and the pea *rbcS* terminator. Next, this construct was inserted into the cloned *PpDRP5B-2* gene together with a hygromycin-resistance (*HPT*) gene (S5 Fig.) because disruption of *PpDRP5B-2* has no effect on *P*. *patens* [15]. PEG-mediated transformation of the ΔB1 knockout line followed, and stable transformants were generated. RT-PCR confirmed the expression of *AtLrgB* in the transformants (S5c Fig.).

### Characterization of *PpLrgB* knockout and *AtLrgB* complemented lines

**Fig 2:**
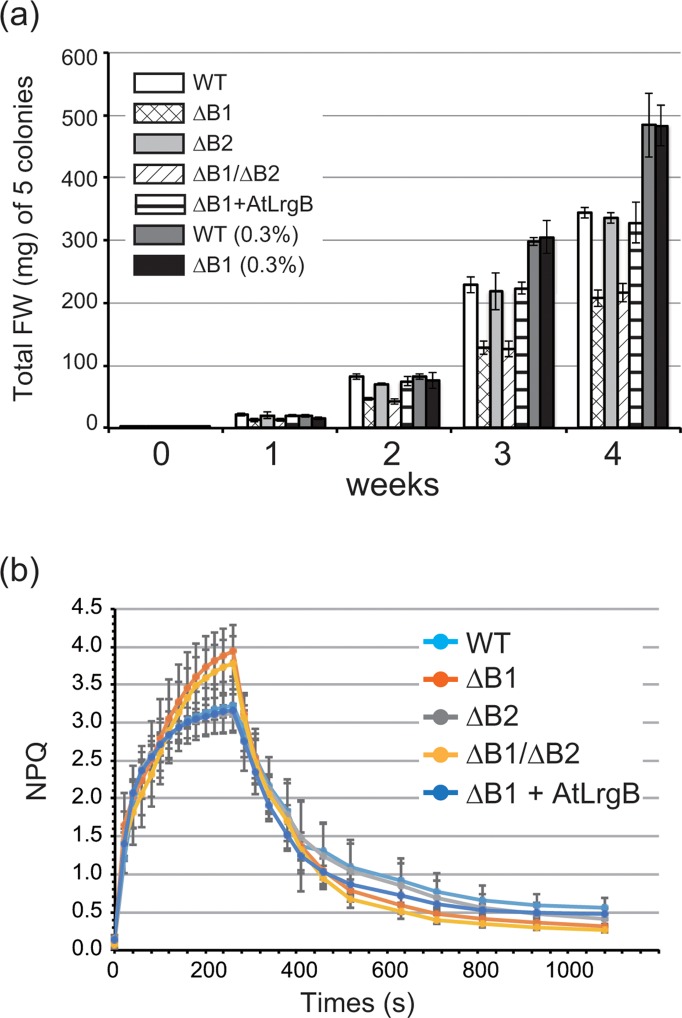
Growth of transformants and NPQ kinetics. (a) Total fresh weights (in mg) of 5 colonies were measured over 4 weeks. The plants studied were the *PpLrgB1* single-(ΔB1)#2, *PpLrgB2* single (ΔB2)#5, and double (ΔB1/ΔB2)#1-knockout lines, and ΔB1 complemented with the *AtLrgB* gene (ΔB1 + AtLrgB)#5. Wild-type (WT) plants were used as controls. Also, data on WT and ΔB1 plants grown under high-CO_2_ conditions are shown as WT (0.3%) and ΔB1 (0.3%), respectively. (b) Nonphotochemical quenching (NPQ) of each line. NPQ kinetics were measured for ΔB1#2, ΔB2#5, ΔB1/ΔB2#1, and ΔB1 + AtLrgB#5 lines in addition to WT plants. Data are presented as means ± SD (n = 6).

During generation of knockout lines lacking each *PpLrgB* gene, we found that protonemal colonies of the ΔB1 line were smaller than those of WT plants (S6 Fig.). Examination of growth confirmed that the ΔB1 line exhibited a lower growth rate than WT plants (Fig. 2a). However, this was not true of the ΔB2 line. Moreover, the ΔB1/ΔB2 line grew at the same rate as the ΔB1 line. These results suggested that *PpLrgB2* knockout did not affect the growth rate. Stable transformation of *AtLrgB* into the ΔB1 line complemented the growth reduction phenotype (Fig. 2, S6 Fig.), suggesting that PpLrgB1 is a plastidic glycolate/glycerate transporter in *P*. *patens*. To confirm the existence of a relationship between the *PpLrgB1* gene and photorespiration, the ΔB1 line was grown under high-CO_2_ [~0.3% (v/v) = 3,000 ppm] conditions. The growth rate of the ΔB1 line increased to that of WT plants under high-CO_2_ conditions (Fig. 2). Although some chlorotic cell death was observed in leaves of the *AtLrgB*-knockout line of *A*. *thaliana* [4,5,6], we did not notice this phenotype in the ΔB1 line, even when cells grown under high-CO_2_ conditions were transferred to ambient air (S7 Fig.).

As mutations in photorespiration-related genes affect photosynthesis in *Arabidopsis* [10], we measured the maximum photochemical efficiencies of PSII [F_v_/F_m_ = (F_m_–F_0_)/F_m_] (Table 1). Although the F_v_/F_m_ value of the ΔB2 line (0.72 for protonemata and 0.76 for gametophores) was unchanged from that of the WT (0.73 for protonemata and 0.77 for gametophores), that of the ΔB1 line decreased slightly to 0.63 for protonemata and 0.73 for gametophores. The value of the ΔB1/ΔB2 line was the same as that of ΔB1. As with the growth rate, the *AtLrgB* gene complemented this phenotype. The F_v_/F_m_ values of ΔB1 cells were normalized under high-CO_2_ conditions (Table 1). These results indicated that the ΔB1 line exhibited a photorespiratory phenotype. *P*. *patens* is known to exhibit high-level NPQ, in contrast to *Arabidopsis* [14]. Our measurements also confirmed high NPQ activity in *P*. *patens* (Fig. 2b). In both the ΔB1 and ΔB1/ΔB2 lines, a increase in NPQ capacity was observed in comparison with other lines, including the WT. The chlorophyll contents of the protonemata were determined for the ΔB1, ΔB2, and ΔB1/ΔB2 mutants, and the ΔB1 mutant complemented with *AtLrgB* (S8 Fig.). The levels of chlorophylls *a* and *b* were unchanged in all mutants.

**Table 1:** F_v_/F_m_ values (*n* = 12).

	WT	ΔB1#2	ΔB2#5	ΔB1/B2#1	ΔB1 +AtLrgB#5	WT CO_2_ 0.3%	ΔB1#2 CO_2_ 0.3%
Protonema	0.73 ± 0.01^a^	0.63 ± 0.02^b^	0.72 ± 0.01^a^	0.64 ± 0.03^b^	0.73 ± 0.02^a^	0.74 ± 0.01^a^	0.72 ± 0.01^a^
Gametophore	0.77 ± 0.01^ab^	0.73 ± 0.01^c^	0.76 ± 0.02^b^	0.72 ± 0.01^c^	0.76 ± 0.01^ab^	0.78 ± 0.01^a^	0.76 ± 0.01^b^

Data were analyzed using the SPSS software by one-way ANOVA followed by the post hoc Tukey test to identify subgroups (a, b and c; *P* < 0.01), indicated by different letters. Data for protonema and gametophores were analyzed separately.

**Fig 3:**
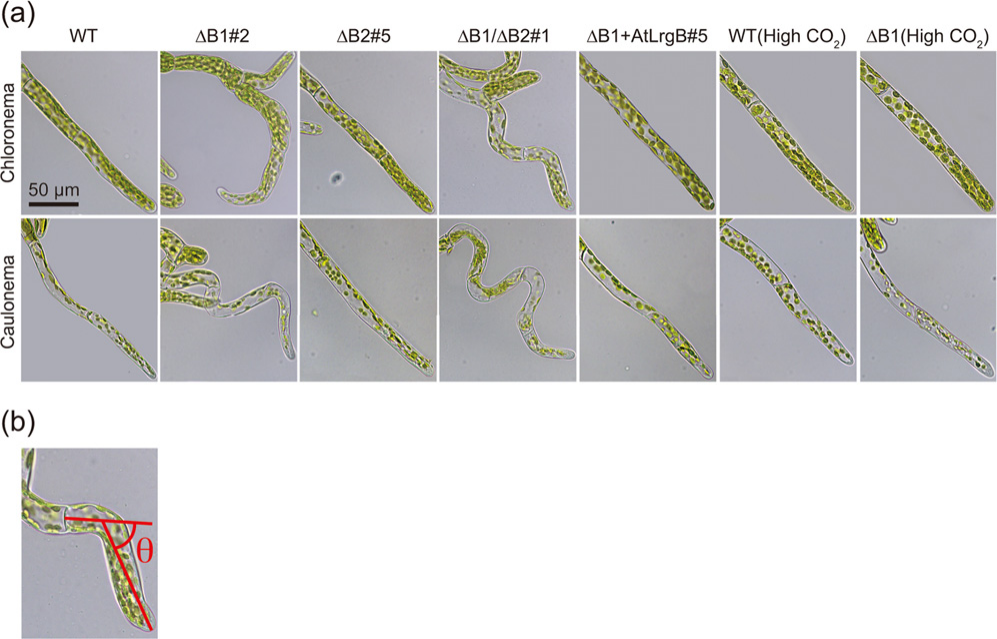
Phenotypes of transformants. (a) Micrographs of chloronema and caulonema cells of the wild type (WT), *PpLrgB1* single-(ΔB1)#2, *PpLrgB2* single (ΔB2)#5 and double (ΔB1/ΔB2)#1-knockout lines, and ΔB1 complemented with the *AtLrgB* gene (ΔB1 + AtLrgB)#5. Chloronema and caulonema cells of WT and ΔB1#2 plants grown under high-CO_2_ conditions for 5 days are also presented. (b) Measurement method for bending angle (θ).

**Fig 4:**
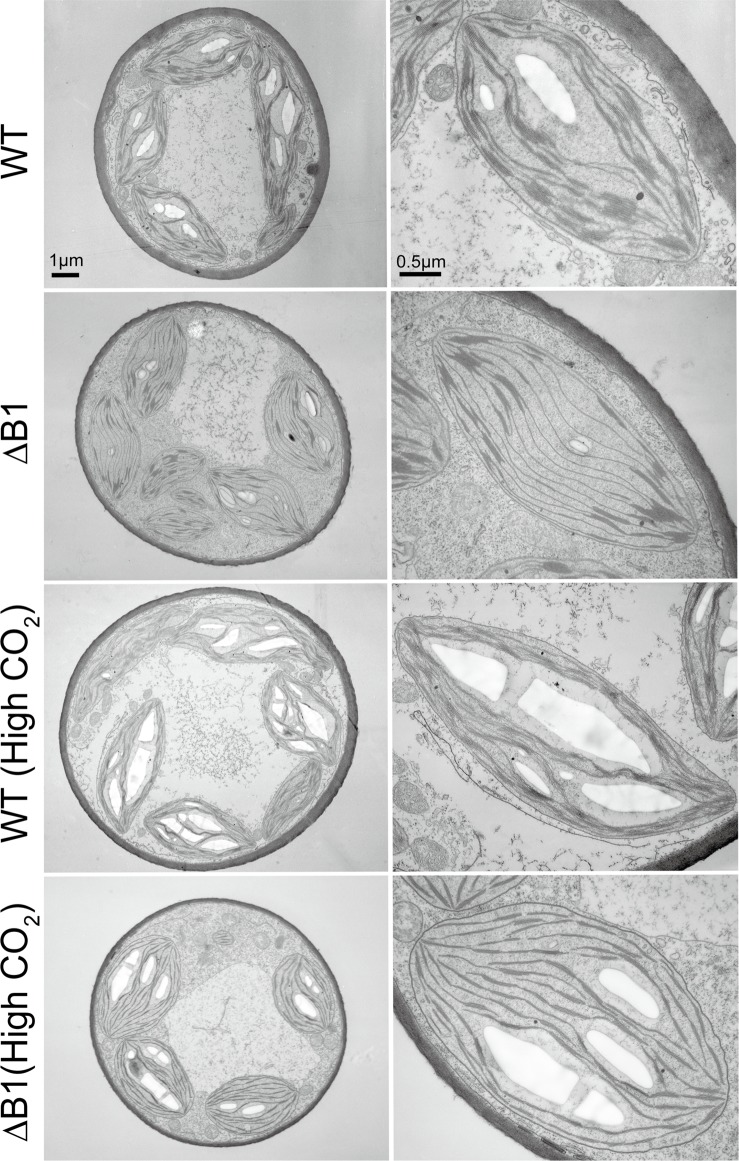
Electron micrographs of the wild type (WT) and *PpLrgB1* knockout (ΔB1) lines. Electron micrographs of protonema cells of WT and ΔB1#2 knockout plants grown in ambient air or under high-CO_2_ conditions are shown. The magnifications in photographs without scale bars are the same as those in the photographs above them.

Microscopic observation showed that both chloronema and caulonema cells of the ΔB1 and ΔB1/ΔB2 mutants were bent, whereas knockout of the *PpLrgB2* gene caused no detectable changes in cell shape (Fig. 3). Measurement of the bending angles of protonemal tip cells confirmed that knockout of the *PpLrgB1* gene caused cells to become curved (Table 2). The *AtLrgB* gene and high-CO_2_ conditions complemented the bending phenotypes (Fig. 3, Table 2). Electron microscopy revealed no obvious difference in the shape of chloroplasts between WT and ΔB1 plants (Fig. 4). However, the ΔB1 plants revealed a significant decrease (level of significance, 1%; t-test) in the number of grana (7.18 ± 1.81 grana/μm^2^) in comparison to wild type plants (9.15 ± 1.60 grana/μm^2^). Chloroplasts both in WT and ΔB1 mutant lines grown under high-CO_2_ conditions had many thylakoids and starch granules.

**Table 2:** Bending angles of protonema tip cells (*n* = 50).

	WT	ΔB1#2	ΔB2#5	ΔB1/B2#1	ΔB1 +AtLrgB#5	WT CO_2_ 0.3%	ΔB1#2 CO_2_ 0.3%
Chloronema	7.9 ± 8.2^a^	31.7 ± 30.6^b^	7.3 ± 7.2^a^	28.5 ± 27.2^b^	8.5 ± 5.9^a^	8.6 ± 5.3^a^	8.3 ± 5.5^a^
Caulonema	18.0 ± 9.9^a^	62.3 ± 23.1^c^	21.4 ± 17.0^a^	63.1 ± 30.5^c^	11.2 ± 5.4^b^	14.7 ± 11.2^ab^	20.5 ± 15.1^a^

Data were analyzed using the SPSS software running the nonparametric Kruskal–Wallis test to identify subgroups (a, b and c; *P* < 0.01). Data for chloronema and caulonema were analyzed separately.

### Glycolate content and effects of photorespiratory metabolites

**Fig 5:**
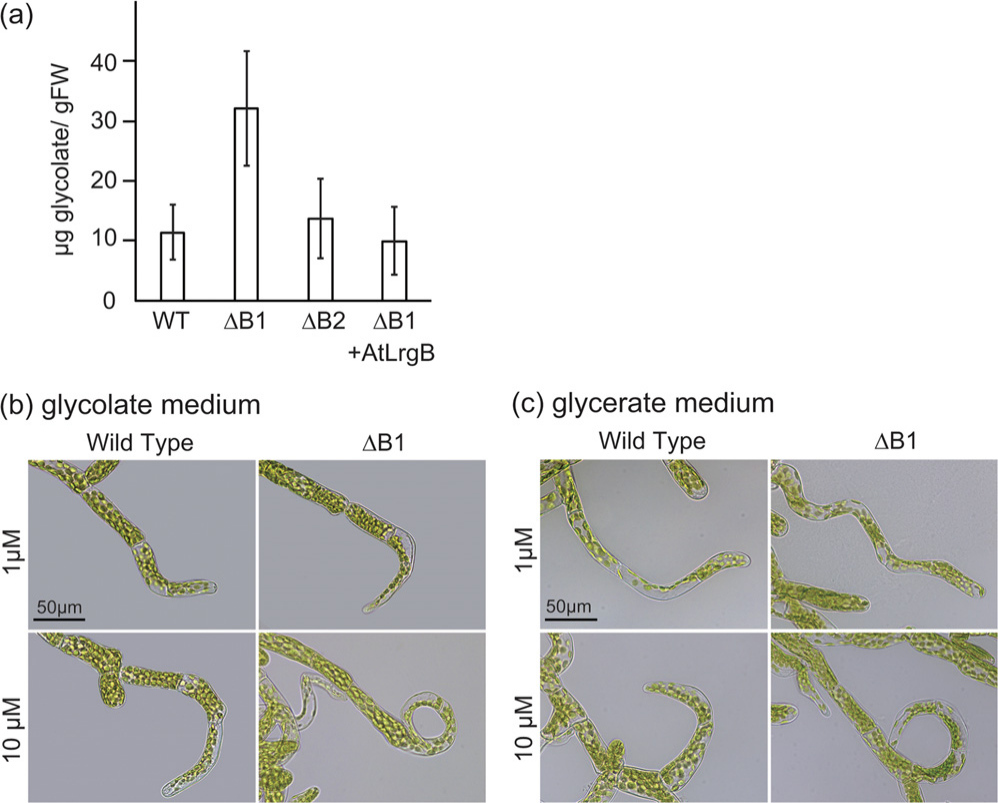
Glycolate contents and effects of glycolate and glycerate on growth. (a) Glycolate levels in wild-type (WT), *PpLrgB1*-knockout (ΔB1)#2, *PpLrgB2*-knockout (ΔB2)#5, and ΔB1 plants complemented with *AtLrgB* #5. (b) Chloronema cells of WT and ΔB1#2 plants grown on media containing glycolate for 5 days are shown. (c) Chloronema cells of WT and ΔB1#2 plants grown on media containing glycerate for 5 days are shown.

If PpLrgB1 were a plastid glycolate/glycerate transporter, glycolate would be expected to accumulate in the ΔB1 line because transport of glycolate from chloroplasts to the cytosol must necessarily be affected by the knockout. We determined that the glycolate content of the ΔB1 line was higher than that of WT cells (Fig. 5a). As expected, glycolate levels were unchanged in the ΔB2 and *AtLrgB* complemented line.

To explore whether bending of protonema cells required accumulation of photorespiratory metabolites, WT and ΔB1 cells were grown on media containing glycolate or glycerate (Fig. 5 and Table 3). Bending of protonema cells of WT plants was evident under such conditions, suggesting that these metabolites caused cell bending.

**Table 3:** Bending angles of protonema tip cells in plants grown on glycolate or glycerate media (*n* = 50).

	WT					ΔB1#2				
Glycolate (μM)	0	1	10	0	0	0	1	10	0	0
Glycerate (μM)	0	0	0	1	10	0	0	0	1	10
Chloronema	7.9 ± 8.2^a^	13.7 ± 16.4^ab^	22.1 ± 18.8^bc^	13.2 ± 13.9^ab^	18.8 ± 16.5^bc^	31.7 ± 30.6^c^	33.3 ± 25.8^c^	33.5 ± 26.7^c^	29.9 ± 21.2^c^	30.0 ± 23.3^c^
Caulonema	18.1 ± 9.9^a^	29.2 ± 20.2^ab^	32.5 ± 18.2^b^	20.2 ± 14.6^a^	23.6 ± 13.9^ab^	62.3 ± 23.1^c^	66.2 ± 33.6^c^	66.9 ± 21.6^c^	58.3 ± 29.7^c^	60.7 ± 31.9^c^

Data were analyzed using the SPSS software running the nonparametric Kruskal–Wallis test to identify subgroups (a, b and c; *P* < 0.01).Data for chloronema and caulonema were analyzed separately.

## Discussion

Our data suggest that PpLrgB1 is a plastid glycolate/glycerate transporter in *P*. *patens*, but the function of PpLrgB2 remains unclear. The *PpLrgB2* gene was expressed, and the gene product was predicted to localize to chloroplasts. Recent proteomic analysis for plastids of *P*. *patens* confirmed the existence of PpLrgB2 in plastids, although PpLrgB1 was not detected [16]. The phenotype of the ΔB2 mutants did not appear to differ from the WT, and the ΔB1/ΔB2 double-knockout lines did not show severe phenotypes in comparison with ΔB1. There is a possibility that PpLrgB2 is a transporter of other nonessential metabolites. At present, we do not exclude the possibility that PpLrgB2 has low glycolate/glycerate transporter activity.

Although ΔB1 plants exhibited decreased growth and photosynthetic activity, we did not observe a cell death phenotype similar to that of *Arabidopsis* mutants. The *atlrgB* mutants showed a chlorotic cell death phenotype upon accumulation of photorespiratory metabolites when grown in ambient air [4,5,6]. In the white sectors of leaves, photoinhibition caused by the loss of transport activity of AtLrgB triggered the cell death cascade. However, as chloroplasts were observed in the green sectors of leaves, the effects of photoinhibition were limited. This may be because photorespiratory metabolites were concentrated to greater than toxic levels in some parts of leaf tissues of *A*. *thaliana*. In contrast, because the protonemata of *P*. *patens* is a filamentous cell layer, diffusion of photorespiratory metabolites such as glycolate and glycerate to the medium may occur and prevent their accumulation in excess of toxic levels. Another reason for the no-cell-death phenotype in *P*. *patens* may be strong NPQ in *P*. *patens* cells using both of the algal-type LHCSR-dependent and PSBS-dependent mechanisms. It is reported that proteins encoded by the *PSBS*, *LHCSR1* and *LHCSR2* genes are all active in NPQ in *P*. *patens* [14]. The triple *psbs lhcsr1 lhcsr2* knockout mutant lacks NPQ, while the double knockout mutant *lhcsr1 lhcsr2* shows the reduction of NPQ related with LHCSR mechanisms [14]. Gene disruption of the *PpLrgB1* gene with these NPQ deficient mutants may be able to demonstrate relationship between photorespiration and NPQ systems. The F_v_/F_m_ value of ΔB1 (Table 1) confirmed that *PpLrgB1* depletion exerted mild effects. Increased NPQ capacity was observed in the ΔB1 line when compared to that of WT (Fig. 2). Decreased photosynthetic activity of ΔB1 must cause reduction of cell growth and colony size. Electron microscopic observation suggested that chloroplasts of the ΔB1 plants had decreased numbers of grana, although the amounts of chlorophyll did not change. Stacked thylakoids were reported to become unstacked under strong illumination to prevent further damage to the D1 protein and facilitate degradation of the photodamaged D1 protein [17]. Therefore, the decrease of grana observed in the ΔB1 plants may be one of the responses to light stress. Measurements of the relative amounts of photosystem I and photosystem II may provide information regarding the phenotypes of thylakoids.

A unique phenotype of the ΔB1 mutant is bending of both cell types of protonema, the chloronemal and caulonemal cells. Protonemal cells of *P*. *patens* have been used as a model system for the study of tip cell growth (reviewed in [18]). At the apical domes of apical cells, polarized secretion of vesicles containing cell wall components and membranes occurs continuously in growing cells, mediated by turgor pressure-driven cell expansion. The cytoskeletons are closely associated with tip growth. In ΔB1 mutants, transition of chloronemata to caulonemata was evident, and both types of protonemal cells expanded in a tip-growing fashion, suggesting that the basal mechanism of tip growth was normal in such cells. The bending angle of caulonemal tip cells was larger than that of chloronemal cells (Table 2). As chloronema cells are chloroplast-rich and grow more slowly than caulonema cells, the higher bending angle of the latter cells may reflect slightly faster growth. As WT cells became bent upon growth on media containing glycolate or glycerate, accumulation of photorespiratory metabolites may explain the bending (Fig. 5 and Table 3), although we cannot exclude the possibility that other non-photorespiratory metabolites generated from glycolate or glycerate cause bending. Glycolate and glycerate are thought to be able to leak through lipid bilayers by slow passive diffusion, as shown for other small organic acids. We advance a hypothesis to explain bending in ΔB1 mutants which involves the functions of acids. Although the pH values of media containing glycolate or glycerate were adjusted to that of normal medium, such acids may weaken the cell wall, compromising straight growth, or may affect the cytoskeleton of apical cells.

The photorespiration system may be conserved in land plants. However, the phenotypes of *LrgB* mutants differ between moss and seed plants. *Arabidopsis* photorespiratory mutants exhibit various phenotypes from severe lethality to minor physiological changes; detailed explanations of mutant-specific phenotypes are as yet unavailable [10]. Accumulation of data on moss photorespiratory mutants via gene knockout in *P*. *patens* may help us to understand the evolution of photorespiration in land plants.

## Methods

### Plant culture

The moss *Physcomitrella patens* Bruch and Schimp. subsp. *patens* strain Cove-NIBB of ecotype Gransden Woods [19] was used as the WT line. Protonemata and gametophores were grown on BCDAT medium solidified with 0.8% (w/v) agar in a chamber at 25ºC under continuous light (40 μmol photon m^–2^ s^–1^; [20]).

For culture under elevated CO_2_ [~0.3% (v/v) = 3,000 ppm] conditions, 17.5 ml of 2 M K_2_CO_3_ and 12.5 ml of 2 M KHCO_3_ were added to a plastic box (ø95 × 110 mm in height) containing an Erlenmeyer flask in which plants were growing, as described by [21]. The solutions were replenished daily. To observe cell death, plants grown for 3 days under high CO_2_ conditions were transferred to ambient air conditions and observed at 2 days after transfer. To observe the effects of glycolate or glycerate on growth, the acids were added at appropriate concentrations to BCDAT medium and the pH adjusted to pH 6.6 with NaOH.

### Characterization of *PpLrgB1* and *B2* genes

The genomic sequence of *P*. *patens* [12] was searched using the tBlastN program and the amino acid sequences encoded by the *AtLrgB* gene of *A*. *thaliana*. We found two genes (Pp1s63_96V6 and Pp1s143_131V6 in the *P*. *patens* genome Ver. 1.6: http://www.phytozome.net) that we termed *PpLrgB1* and *PpLrgB2*, respectively. A phylogenetic tree was constructed using the MEGA5 software [22].

RNA was isolated from WT protonemata by a method described previously [23]. Each cDNA was amplified by RT-PCR using the primer sets PpLrgB1/F0-New and PpLrgB1/R0 for PpLrgB1, or PpLrgB2/F0 and PpLrgB2/R0 for PpLrgB2. Probes for Northern hybridization were generated using a PCR DIG Probe Synthesis Kit (Roche Diagnostics) and the appropriate primer sets, PpLrgB1/F1 and PpLrgB1/R0 for PpLrgB1, or PpLrgB2/F2 and PpLrgB2/R0 for PpLrgB2. The primers used are listed in the S9 Fig.

### Subcellular localization of PpLrgB-GFP fusion proteins

Computer predictions of protein subcellular localizations were obtained using the TargetP program [24]. To construct the PpLrgB1(TP)-GFP plasmid, in which GFP was fused to the N-terminus of PpLrgB1 driven by the CaMV 35S promoter, we used a DNA fragment that included sequence coding for the N-terminal 82 amino acid residues, amplified via genomic PCR using the PpLrgB1-F0-SalI and PpLrgB1-R3-SalI primers. This DNA was digested with *Sal*I to cut restriction sites in primers and inserted into the *Sal*I-digested sGFP(S65T) plasmid [25]. *P*. *patens* was transformed as described previously [23]. The PpLrgB2(TP)-GFP plasmid contained the N-terminal 91 amino acid residues of PpLrgB2.

### Generation of knockout lines

Plasmid pTN3 carrying the *NPTII* gene [20] was used to target *PpLrgB1* (S4 Fig.). The *NPTII* gene cassette consisted of the CaMV 35S promoter, the neomycin phosphotransferase gene, and the CaMV 35S polyadenylation sequence. Genomic DNA was isolated from the protonemata of *P*. *patens* using the cetyltrimethylammonium bromide (CTAB) method [23]. The 5′ untranslated region, and the first exon and first intron of *PpLrgB1*, were amplified from genomic DNA via PCR using PpLrgB1-F4 and PpLrgB1-R1 primers; subjected to blunting using a TaKaRa DNA blunting Kit (TaKaRa Bio); and cloned into the blunted *Eco*RI site located upstream of the *NPTII* gene cassette of pTN3. Next, the 3′ untranslated region was PCR-amplified using the PpLrgB1-F5 and PpLrgB1-R2 primers, subjected to blunting, and inserted into the blunted *Bam*HI site located downstream of the *NPTII* gene cassette. The plasmid thus constructed was linearized by digestion with *Kpn*I and *Sac*I, and used to transform *P*. *patens*. Primary screening of transformants was carried out by genomic PCR using the gene-specific primer and a primer specific for the *NTPII* gene cassette. Southern hybridization was used to detect additional insertions of transformed DNA into the *P*. *patens* genome (S4 Fig.). Probes for Southern hybridization were generated using the PCR DIG Probe Kit (Roche Diagnostics) employing PpLrgB1-F7 and PpLrgB1-R3 primers. The transcribed region of the *PpLrgB1* gene had one *Hin*dIII and no *Eco*RV site, while the insert DNA contained no *Hin*dIII site and one *Eco*RV site. Therefore, the sizes of hybridizing bands for transformed genomic DNAs changed from 5.7 (WT) to 10.3 kbp for the *Hin*dIII restriction pattern and from 10.7 (WT) to 4.3 kbp for the *Eco*RV restriction pattern when the plasmid for gene disruption was inserted into the *PpLrgB1* gene region (S4 Fig.). If transformants had additional insertions, other hybridized bands were observed. We selected four transformants with cell-bending phenotypes from the primary screened lines and determined that only line #2 had no additional insertions (S4 Fig.).

The p35S-Zeo plasmid carrying a zeocin-resistance gene expression cassette [26] was used to target *PpLrgB2* (S4 Fig.). The 3′ region of *PpLrgB2* was amplified by genomic PCR with the PpLrgB2-F7 and PpLrgB2-R3 primers, subjected to blunting, and inserted into the blunted *Hin*dIII site of the p35S-Zeo plasmid. Next, the 5′ region of the *PpLrgB2* gene was amplified with the PpLrgB2-F5 and PpLrgB2-R1 primers, subjected to blunting, and inserted into the blunted *Xba*I site. The constructed plasmid was linearized by digestion with *Kpn*I and *Sac*I, and used to transform wild-type *P*. *patens* plants. For generation of *PpLrgB1/B2* double-knockout lines, we selected *PpLrgB1*-knockout line #2 (S4 Fig.). Primary screening of transformants was carried out by genomic PCR using the gene-specific primer and a primer specific for the zeocin gene cassette. The insert DNA copy number was determined by Southern hybridization (S4 Fig.). The transcribed region of the *PpLrgB2* gene contained one *Hin*dIII site and one *Eco*O109I site, while the insert DNA contained one *Hin*dIII site in the 5′ untranscribed region of the *PpLrgB2* gene and one *Eco*O109I site. Therefore, sizes of the hybridizing bands for transformed genomic DNAs changed from 4.6 (WT) to 6.3 kbp for the *Hin*dIII restriction pattern and from 5.0 (WT) to 4.4 kbp for the *Eco*0109I restriction pattern when the plasmid for gene disruption was inserted into the *PpLrgB2* gene region (S4 Fig.). If transformants had additional insertions, other hybridized bands were observed. We selected several transformants from the primary screened lines and determined that lines #1 and #5 for ΔB2, and lines #1 and #9 for ΔB1/ΔB2 had no other insertions in the transformant genomes. For RT-PCR, we isolated RNAs from mutant and WT plants, treated the RNAs with DNase I, and used the RNAs to generate cDNA from oligo-dT primers. RT-PCR was performed using appropriate primer sets: PpLrgB1/F0-New and PpLrgB1/R0 for PpLrgB1, or PpLrgB2/F0 and PpLrgB2/R0 for PpLrgB2. *PpActin* served as a control.

### Generation of *AtLrgB* complemented lines

For cross-species complementation testing, we used the *AtLrgB* gene of *A*. *thaliana*. *AtLrgB* cDNA was amplified by RT-PCR from DNaseI-treated total RNA of *A*. *thaliana* using the AtLrgB-F0 and AtLrgB-R0 primers, and cloned into the pBluescript vector (Agilent Technologies). The cDNA region was extracted by digestion with *Eco*RI and *Bam*HI, blunted, and inserted at the *Eco*RV site between the rice actin promoter and the pea *rbcS* terminator of the pTKM1 [27] plasmid. We used the *P*. *patens* dynamin-related protein 5B-2 (*PpDRP5B-2*) genomic region for complementation analysis because disruption of the *PpDRP5B-2* gene did not visibly affect *P*. *patens* [15]. The hygromycin phosphotransferase (*HPT*) gene was inserted into the *Eco*RV fragment region of the cloned *PpDRP5B-2* gene [28]. *AtLrgB* cDNA, with the promoter and terminator, was isolated by digestion with *Xba*I and *Kpn*I, subjected to blunting, and inserted into the *Nhe*I site of the cloned *PpDRP5B-2* gene bearing the *HPT* gene; *P*. *patens* transformation followed (S5 Fig.). PEG-mediated transformation was performed using *PpLrgB1* knockout line #2. Insertion of *AtLrgB* cDNA in the *PpDRP5B-2* region, and expression thereof, were confirmed by Southern and RT-PCR analyses (S5 Fig.).

### Characterization of the *PpLrgB* knockout and *AtLrgB* complemented lines

To measure protonemal growth, we selected five small colonies of similar sizes and adjusted the total weight to 2.5 μg/5 colonies. We used one group of 5 colonies for each point in time. Each colony was transferred to fresh BCDAT medium. One group of 5 colonies was chosen at the 1, 2, 3 and 4 weeks after transfer, and the total fresh weights of five colonies in the group were measured. Measurements were repeated five times.

We used a PAM-2500 Chlorophyll Fluorometer (Walz) to measure chlorophyll fluorescence parameters. Protonemata grown for 4–5 days, gametophores grown for 4 weeks, or gametophores grown for 3 weeks under high-CO_2_ conditions were used. Samples were dark-treated for 30 min and next subjected to measurements. The intensities of saturating and actinic light were 4,000 and 828 μmol photon m^–2^ s^–1^, respectively. F_v_/F_m_ and NPQ parameters were calculated as (F_m_–F_0_)/F_m_ and (F_m_–F_m_′)/F_m_′, respectively.

Chlorophyll was extracted into 80% (v/v) acetone from the protonemata of WT plants, knockout mutants, and ΔB1 plants complemented with the *AtLrgB* gene after 5 days growth in fresh medium. Measurements were repeated three times. Chlorophyll contents were measured using a Gene Spec III Spectrophotometer (Hitachi High-Technologies) and calculated using the following formulae: chlorophyll *a* = [(12.7 × A_663_)–(2.6 × A_645_) × ml acetone/sample fresh weight (mg)] and chlorophyll *b* = [(22.9 × A_645_)–(4.68 × A_663_) × ml acetone/sample fresh weight (mg)] [29].

To measure the bending angles of the protonemata (Fig. 3), cells cultured for 4–5 days after transfer to new medium were used. First, the locus exhibiting the most bending was determined in a tip cell. The center of the width was positioned at this point. From this position, we drew two lines to the centers of the tip and bottom of the cell in the longitudinal direction and measured bending angles using the Angle Measurement Function of AxioVision (Zeiss).

To measure glycolate contents, the protonemata were cultured in liquid BCDAT medium with aeration for 1 week. Glycolate contents were measured using the quantitative 2,7-dihydroxynaphthalene colorimetric method [30]. To observe the effects of photorespiratory metabolites, protonema cells were cultured on BCDAT media containing glycolate or glycerate (1 μM or 10 μM) for 5 days.

### Microscopic observations

Bright-field and epifluorescent cell images were recorded using a charge-coupled device (CCD) camera (Zeiss Axiocam) fitted to a microscope with filter sets for FITC and rhodamine 123 (Zeiss Axioskop 2 plus). For electron microscopy, samples were fixed in 2% glutaraldehyde buffered with 50 mM sodium cacodylate (pH 7.4), exposed to a 2% osmium tetroxide aqueous solution containing 0.1% potassium hexacyanoferrate (II), dehydrated through a graded ethanol series, and embedded in Quetol-651 resin. Thin sections were cut and stained with uranyl acetate and lead citrate, and observed using a JEM-1200EX transmission electron microscope (JEOL). To show the differences between chloroplasts of wild-type and ΔB1 plants, the number of grana and size of chloroplasts was measured for 20 chloroplast sections. The stacked thylakoids with two layers were recognized as grana.

## Supporting Information

S1 Fig Comparison of the amino-acid sequences encoded by the *PpLrgB1* (B1) and *LrgB2* (B2) genes with those of *A. thaliana* (At) and *E. coli* (Ec). Sequence number is shown on the right. Predicted cutting sites for the transit peptide are indicated by *triangles*. Amino acids identical in all sequences are indicated by *blue boxes*, and amino acids identical in all plant sequences are indicated by *purple boxes*.
(EPS)

S2 Fig Phylogenetic relationships among LrgB proteins. Evolutionary history was inferred using the neighbor-joining method [31]. The optimal tree is shown. The percentages of replicate trees in which the associated taxa clustered together in the bootstrap test (1,000 replicates) are given next to the branches [32]. The tree is drawn to scale, and the branch lengths are in the same units employed to describe the evolutionary distances used to infer the phylogenetic tree. Evolutionary distances were computed using the p-distance method [33], and the unit is the number of amino acid differences per site. Analysis featured 61 amino acid sequences. All ambiguous positions were removed for each sequence pair. The final data set contained a total of 649 positions.
(EPS)

S3 Fig Expression of *P. patens LrgB* genes in the wild-type (WT) and knockout lines. (a) Northern blot analysis using each gene as a probe. Methylene Blue staining of rRNA bands was used as a control. (b) The expression levels of each gene in both WT and knockout plants (ΔB1#2, ΔB2#5 and ΔB1/ΔB2#1) were determined via RT-PCR. The *PpActin* gene was used as an internal control.
(EPS)

S4 Fig Generation of *PpLrgB*-knockout lines. (a) Schematic representation of the *PpLrgB1* genomic region in wild-type (WT, top) and knockout (KO, bottom) plants. The plasmid constructed for gene disruption is shown in the middle with pTN3 vector sequences omitted. Exons are indicated by black boxes. The probe region and the predicted sizes of restriction fragments detected in the Southern blot analyses are given. The *NPTII* gene cassette consisted of the CaMV 35S promoter (P35), the neomycin phosphotransferase gene (*NPTII*), and the CaMV 35S polyadenylation sequence (35PA). (b) Schematic representation of the construction of *PpLrgB2*-knockout lines. (c) Southern blot hybridization data derived using the *PpLrgB1* probe are shown. Genomic DNAs from the WT and *PpLrgB1* knockout line #2 were digested with *Hin*dIII or *Eco*RV. Other data have been removed from the photograph. (d) Southern blot hybridization data derived using the *PpLrgB2* probe are shown. Genomic DNAs from the WT, and *PpLrgB2* knockout lines #1 and #5, were digested with *Eco*O109I or *Hin*dIII. (e) Southern blot analysis of double-knockout lines using the *PpLrgB2* probe. *PpLrgB1*-knockout line #2 was used to generate the *PpLrgB1/B2* double-knockout lines.
(TIF)

S5 Fig Generation of lines in which a *PpLrgB1* deletion was complemented with the *AtLrgB* gene. (a) Schematic representation of the *PpDRP5B-2* genomic regions in the *PpLrgB1*-knockout line #2 (top) and the *AtLrgB* complemented (bottom) line. The plasmid constructed for complementation is shown in the middle. Exons are indicated by black boxes. The probe region and predicted sizes of restriction fragments detected in Southern blot analyses are given. Act1P, rice actin promoter; *rbcT*, pea *rbcS* terminator; HPT, hygromycin phosphotransferase gene. (b) Southern blot hybridization analysis using the *PpDRP5B-2* probe. Genomic DNAs from wild-type (WT) and *AtLrgB* complemented plants #5, #6, #7, #11, #17, #18, and #19 were digested with *Hin*dIII or *Xba*I. (c) RT-PCR data derived using *AtLrgB* primers are shown. Primer locations are indicated in (a). The *PpActin* gene was used as an internal control.
(TIF)

S6 Fig Photos of 4-week cultured colonies. The plants studied were the *PpLrgB1* single-(ΔB1)#2, *PpLrgB2* single (ΔB2)#5, and double (ΔB1/ΔB2)#1-knockout lines and the ΔB1 line complemented with the *AtLrgB* gene (ΔB1 + AtLrgB)#5.
(EPS)

S7 Fig Observation of protonemal cells of the *PpLrgB1*-knockout line #2 after transfer to ambient air from high CO_2_ conditions. (TIF)

S8 Fig Chlorophyll contents of WT, ΔB1#2, ΔB2#5, ΔB1/ΔB2#1, and ΔB1 plants complemented with *AtLrgB* (ΔB1+AtLrgB)#5. (EPS)

S9 Fig Primers used in this study. (EPS)
