# Supplementary figures and images for: Bending of Protonema Cells in a Plastid Glycolate/Glycerate Transporter Knockout Line of Physcomitrella patens
Source: PLoS One. 2015 Mar 20;10(3):e0118804. doi: 10.1371/journal.pone.0118804 (PMC4368765; doi:10.1371/journal.pone.0118804)

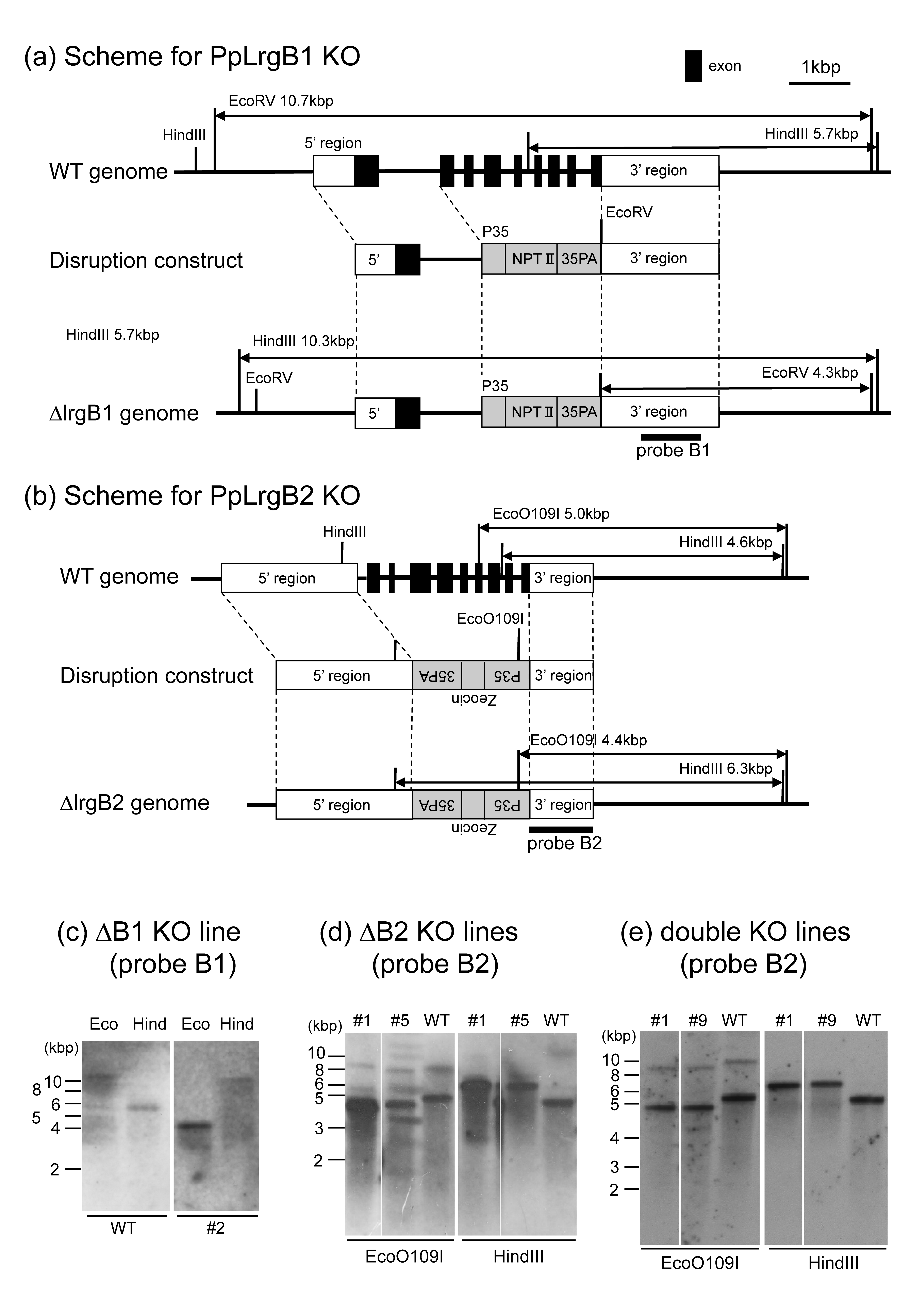

Supplement: S4 Fig — (a) Schematic representation of the PpLrgB1 genomic region in wild-type (WT, top) and knockout (KO, bottom) plants. The plasmid constructed for gene disruption is shown in the middle with pTN3 vector sequences omitted. Exons are indicated by black boxes. The probe region and the predicted sizes of restriction fragments detected in the Southern blot analyses are given. The NPTII gene cassette consisted of the CaMV 35S promoter (P35), the neomycin phosphotransferase gene (NPTII), and the CaMV 35S polyadenylation sequence (35PA). (b) Schematic representation of the construction of PpLrgB2-knockout lines. (c) Southern blot hybridization data derived using the PpLrgB1 probe are shown. Genomic DNAs from the WT and PpLrgB1 knockout line #2 were digested with HindIII or EcoRV. Other data have been removed from the photograph. (d) Southern blot hybridization data derived using the PpLrgB2 probe are shown. Genomic DNAs from the WT, and PpLrgB2 knockout lines #1 and #5, were digested with EcoO109I or HindIII. (e) Southern blot analysis of double-knockout lines using the PpLrgB2 probe. PpLrgB1-knockout line #2 was used to generate the PpLrgB1/B2 double-knockout lines. (TIF) [file pone.0118804.s004.tif]

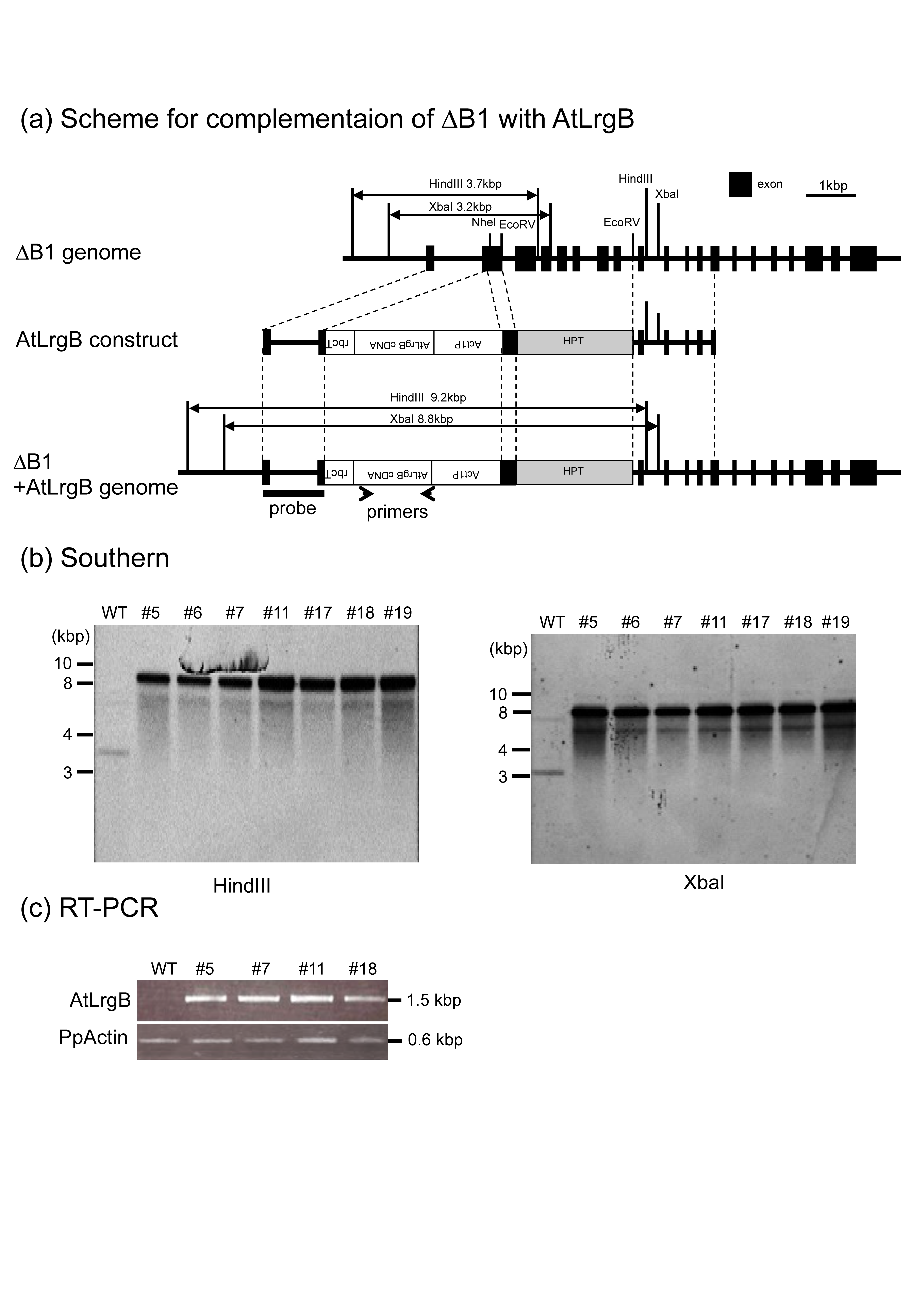

Supplement: S5 Fig — (a) Schematic representation of the PpDRP5B-2 genomic regions in the PpLrgB1-knockout line #2 (top) and the AtLrgB complemented (bottom) line. The plasmid constructed for complementation is shown in the middle. Exons are indicated by black boxes. The probe region and predicted sizes of restriction fragments detected in Southern blot analyses are given. Act1P, rice actin promoter; rbcT, pea rbcS terminator; HPT, hygromycin phosphotransferase gene. (b) Southern blot hybridization analysis using the PpDRP5B-2 probe. Genomic DNAs from wild-type (WT) and AtLrgB complemented plants #5, #6, #7, #11, #17, #18, and #19 were digested with HindIII or XbaI. (c) RT-PCR data derived using AtLrgB primers are shown. Primer locations are indicated in (a). The PpActin gene was used as an internal control. (TIF) [file pone.0118804.s005.tif]

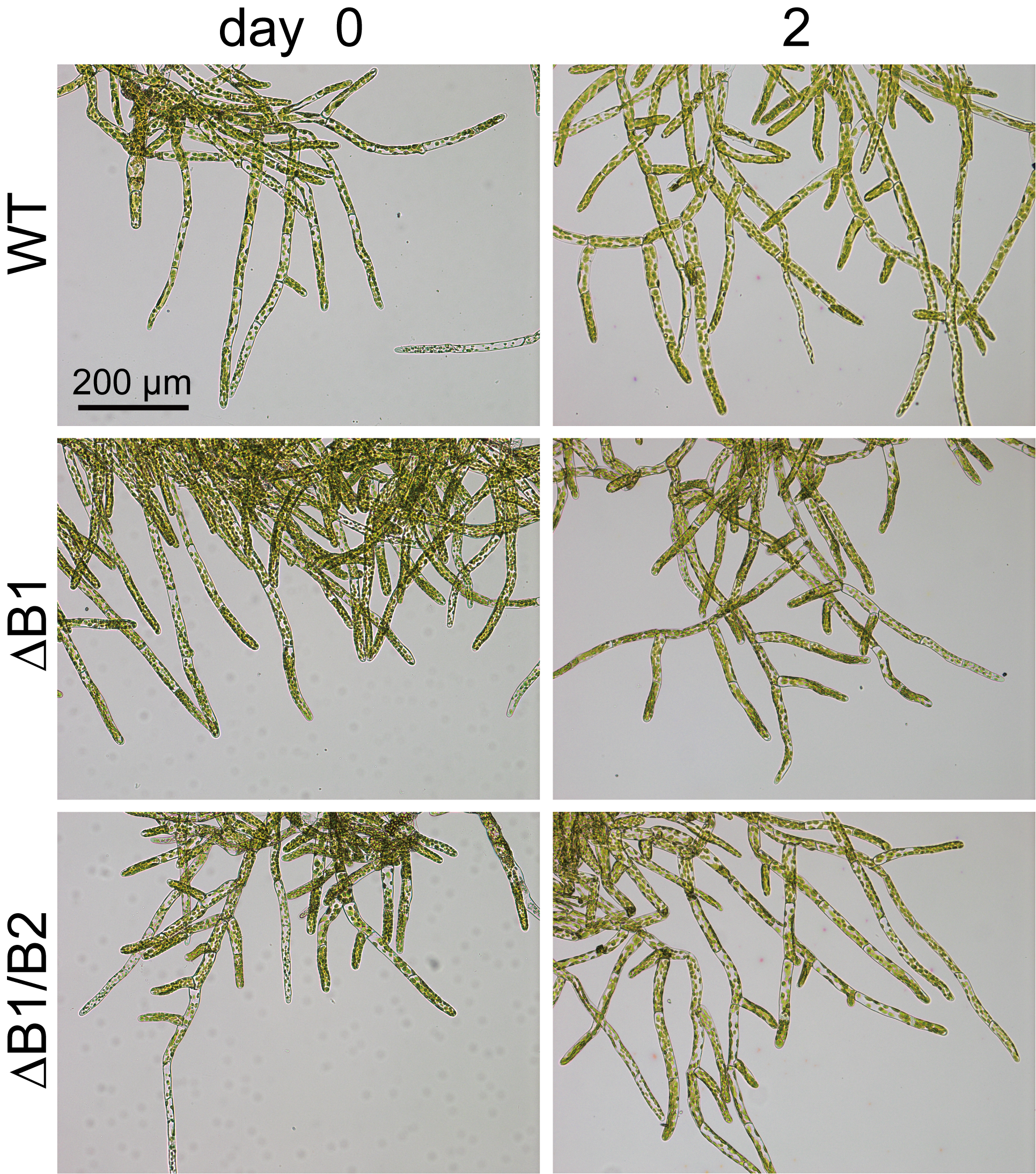

Supplement: S7 Fig — (TIF) [file pone.0118804.s007.tif]
